# Prospective Multicenter Surveillance of Non–*H*. *pylori Helicobacter* Infections during Medical Checkups, Japan

**DOI:** 10.3201/eid3106.241315

**Published:** 2025-06

**Authors:** Kengo Tokunaga, Emiko Rimbara, Toshihisa Tsukadaira, Katsuhiro Mabe, Koji Yahara, Hidekazu Suzuki, Tadashi Shimoyama, Mitsushige Sugimoto, Tadayoshi Okimoto, Hidenori Matsui, Masato Suzuki, Keigo Shibayama, Hiroyoshi Ota, Kazunari Murakami, Mototsugu Kato

**Affiliations:** Kyorin University School of Medicine, Tokyo, Japan (K. Tokunaga); National Institute of Infectious Diseases, Tokyo (E. Rimbara, K. Yahara, H. Matsui, M. Suzuki); Kenwakai Hospital, Iida, Japan (T. Tsukadaira); Mabe Goryokaku Gastrointestinal Endoscopy Clinic, Hakodate, Japan (K. Mabe); Tokai University School of Medicine, Isehara, Japan (H. Suzuki); Aomori General Health Examination Center, Aomori, Japan (T. Shimoyama); Oita University, Oita, Japan (M. Sugimoto, K. Murakami); Oita Prefectural Hospital, Oita (T. Okimoto); Nagoya University Graduate School of Medicine, Nagoya, Japan (K. Shibayama); Shinshu University School of Medicine, Matsumoto, Japan (H. Ota); Public Interest Foundation Hokkaido Cancer Society, Sapporo, Japan (M. Kato)

**Keywords:** *Helicobacter* infections, *Helicobacter suis*, *Helicobacter pylori*, NHPH, non–*Helicobacter pylori Helicobacter*, gastric endoscopy, gastric diseases, bacteria, enteric infectons, Japan

## Abstract

To evaluate non–*H. pylori Helicobacter* (NHPH) infections in Japan, we enrolled 673 consecutive patients who underwent gastric endoscopy during annual medical checkups at 4 hospitals during April 2022–February 2023. We collected intragastric fluid and serum samples to detect NHPH infection by PCR and serologic tests. The prevalence of NHPH was 3% (20/673); 70% (14/20) of patients were infected with *H. suis* and 30% (6/20) with non–*H. suis* NHPH species. All 14 *H. suis*–infected patients were men and had a history of pork offal ingestion. Among non–*H. suis* NHPH-infected patients, 50% (3/6) owned pet cats, whereas only 22% (145/667) of other patients owned cats. Endoscopic evaluation revealed marbled crack-like gastritis was present in 93% (13/14) of *H. suis*–infected patients, a significantly higher prevalence than for *H. pylori*–infected (28.6%) and *H. pylori* eradication therapy (27.6%) groups. Pork offal ingestion and having pet cats increase risk for *Helicobacter* spp. infections.

Non–*H. pylori Helicobacter* (NHPH) species are helical corkscrew-like bacteria; *Helicobacter* spp. have been reported since the 1980s to infect the human stomach (*1*). In humans, NHPH infection causes gastric diseases, such as peptic ulcers, chronic gastritis, gastric cancer, and gastric mucosa-associated lymphoid tissue (MALT) lymphoma (*2*–*5*). Although *H. suis* is the most common NHPH species that infects the human stomach (*4*,*6*,*7*), its pathogenicity remains unclear because isolation has not previously been feasible. The recent successful isolation of *H. suis* from patients with gastric disease demonstrated *H. suis* pathogenicity in the human stomach (*8*), ushering in a new stage of NHPH research.

NHPH infection rates have mostly been analyzed in patients with specific gastric-related diseases (*2*–*5*,*9*–*12*). Most studies have used gastric biopsy to detect NHPH infection, making it difficult from an ethical standpoint to investigate NHPH infection in asymptomatic persons. Recent studies have shown the usefulness of gastric juice samples for *H. pylori* diagnoses (*13*,*14*), and we recently developed a method to detect NHPH infections by using gastric juice samples (*15*,*16*) without performing a biopsy. In addition, we developed a highly sensitive serologic diagnostic method for *H. suis* infection in humans (*15*,*16*). We conducted a multicenter survey of NHPH infection among patients who underwent gastric endoscopy during medical checkups in Japan by using those methods. We evaluated NHPH infection rates and regional differences, identified bacterial species, and determined associations between NHPH stomach infections, demographics, and endoscopic findings. 

## Materials and Methods

### Ethics

We explained the study to all participants and obtained written informed consent from each. The study was approved by the Ethics Committees of Kyorin University Hospital, Junpukai Health Maintenance Center (Kurashiki, Japan), Kenwakai Hospital, and Hokkaido Cancer Society (approval no. 810, project no. R03-052-52) and registered with the University Hospital Medical Information Network, Clinical Trials Registry (UMIN registration no. UMIN000054538).

### Study Design, Inclusion Criteria, and Sample Collection

**Figure 1:**
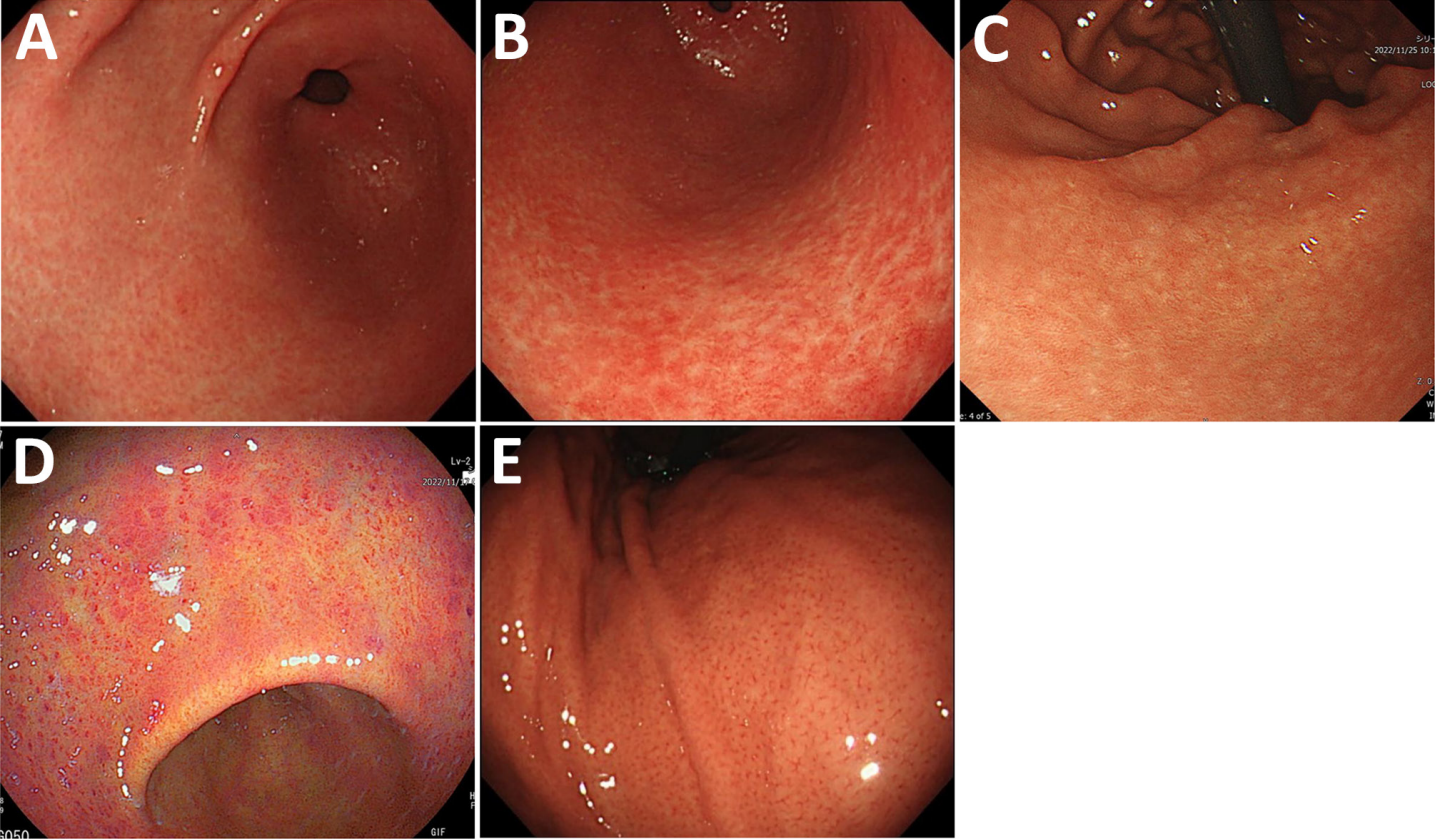
Representative endoscopic images of gastritis types observed in study of non–*H. pylori* *Helicobacter* infections, Japan, 2022. A) White marbled appearance. A white mesh pattern composed of shallow depressions that looks like white marble from the gastric antrum to the angle. White light imaging (WLI). B) Crack-like mucosa. A mesh pattern composed of faded and depressed lines with varied widths on coarse surfaces (WLI). C) Nodular gastritis. Compared with nodular gastritis caused by *H. pylori* infection, nodules that are shorter and appear as white spots are also considered nodular gastritis (WLI). D) Spotty redness, includes findings similar to those of *H. pylori* infection in the gastric body and the antrum (linked-color imaging). E) Regular arrangement of collecting venules. Microvessels with starfish-like appearance are observed as minute red points in the gastric lower body (WLI).

We enrolled consecutive patients who underwent an upper endoscopy during annual medical checkups at 4 hospitals in Japan during April 2022–February 2023. Hospital A is in Hokkaido, hospital B is in Tokyo, hospital C is in Nagano, and hospital D is in Okayama. We excluded patients who underwent gastrectomy. We collected gastric washes (intragastric fluid) and serum samples from all patients to test for NHPH infection by PCR (fluids) and for *H. suis* by ELISA (serum samples). We collected the following clinical information: age; sex; endoscopic findings; and histories of *H. pylori* infection, *H. pylori* eradication therapy, pork offal ingestion, and pet ownership. We assessed the endoscopic grade of gastric mucosal atrophy by using the Kimura and Takemoto classification (*17*). To verify the endoscopic characteristics of NHPH infection, we confirmed the presence or absence of white-marbled appearance (*18*) and crack-like mucosa (*11*). We defined white-marbled appearance as a mottled mucosa with a white mesh pattern from the gastric antrum to the angulus (Figure 1, panel A; Appendix Figure 1) and crack-like mucosa as a mesh pattern composed of faded, depressed, and varying width lines on a coarse surface from the gastric antrum to angulus (Figure 1, panel B; Appendix Figure 1). Although both appearances are similar to atrophy, they have a white mesh; however, the crack-like mucosa appearance also has depressions in the white mesh. We defined a white-marbled with crack-like mucosa appearance as marbled crack-like (MARC) gastritis. In NHPH-infected patients, we also examined the presence and location of nodular gastritis from the antrum to angulus (Figure 1, panel C), spotty redness from the antrum to the body (Figure 1, panel D), and the regular arrangement of collecting venules (RAC; Figure 1, panel E) (*19*). We defined endoscopic findings as normal if no atrophic gastritis, peptic ulcers, gastric cancer, nodular gastritis, white-marble appearance, or crack-like mucosal gastritis were present.

We divided *H. pylori* infections into the following 3 groups: infected with *H. pylori* (*H. pylori* infection group), history of *H. pylori* infection treated with eradication therapy (posteradication group), and no history of *H. pylori* infection (no *H. pylori* infection group). Diagnosis of *H. pylori* infection was confirmed by endoscopy physicians (K.T., K.M., T.K., and M.K.) with >10 years of experience in diagnosing *H. pylori* infection, according to results of a urea breath test, serum *H. pylori* antibody test, fecal antigen test, rapid urease test, histologic studies, and endoscopic observation, according to the Kyoto classification of gastritis (*20*). The posteradication group also included patients with no history of *H. pylori* eradication but with an endoscopically suspected history of *H. pylori* infection, according to the Kyoto classification of gastritis.

### NHPH Detection by PCR and ELISA

We detected NHPH infection by PCR of DNA extracted from intragastric fluid collected during endoscopic examination. We centrifuged 1 mL of intragastric fluid and collected the precipitates for DNA extraction by using the DNeasy Blood and Tissue Kit (QIAGEN, https://www.qiagen.com). We performed PCR by using NHPH species–specific primers targeting 16S rRNA and *H. suis*–specific primers targeting the *hsvA* gene, as previously described (*8*). We determined the samples to be *H. suis* positive by using both PCR primer sets. However, if PCR targeting 16S rRNA for NHPH species was positive and PCR targeting *hsvA* was negative, we identified those samples as non–*H. suis* NHPH positive.

We measured serum *H. suis* antibody titers as previously described (*15*,*16*). In brief, we determined *H. suis* antibody titers by using ELISA and calculated the cutoff index (COI) value as the ratio of the absorbance of the sample to the absorbance of the positive control, both at 450 nm. We deemed the samples as positive when COI values were >1.

### Statistics

We examined associations between clinical characteristics of patients and the specific status of *Helicobacter* infection (i.e., infection by *H. suis*, non–*H. suis* NHPH, or *H. pylori*, as well as after *H. pylori* eradication and absence of *Helicobacter* infection without eradication therapy) by using the Fisher exact test for discrete characteristics and Wilcoxon rank-sum test for continuous characteristics. We also examined the association between specific hospitals and *Helicobacter* infection status by using the Fisher exact test. We evaluated differences in age and *H. suis* ELISA titers between groups by using the Kruskal-Wallis test. We used SPSS Statistics 29 (IBM, https://www.ibm.com) and R version 4.3.2 (The R Project for Statistical Computing, https://www.r-project.org) for statistical analyses and set statistical significance at p<0.05.

## Results

### Prevalence of NHPH Infection

Five patients were excluded from the analysis; 2 at hospital B and 1 at hospital D underwent remnant gastrectomy, and intragastric fluid or serum could not be obtained from 2 other patients at hospital B. We included 673 participants in the study: 100 at hospital A, 198 at hospital B, 199 at hospital C, and 179 at hospital D. The median patient age was 55 (range 29–91) years; 57.6% were male and 42.4% female (Table 1).

**Table 1:** Characteristics of patients in study of non–*H. pylori* *Helicobacter* infections, Japan, 2022*

Characteristics	Total, n = 673	Hospital			
		A, n = 100	B, n = 198	C, n = 199	D, n = 176
Median age, y (range)	55 (29–91)	64 (43–88)	61 (29–91)	52 (31–77)	51 (34–77)
Sex					
M	390 (57.9)	47 (47.0)	119 (60.1)	111 (55.8)	113 (64.2)
F	283 (42.1)	53 (53.0)	79 (39.9)	88 (44.2)	63 (35.8)
Endoscopic findings					
Normal	322 (47.8)	56 (56.0)	114 (57.6)	73 (36.7)	79 (44.9)
Atrophic gastritis	340 (50.5)	41 (41.0)	80 (40.4)	125 (62.8)	94 (53.4)
Peptic ulcer	5 (0.7)	0	2 (1.0)	0	3 (1.7)
Gastric cancer	7 (1.0)	0	1 (0.5)	0	6 (3.4)
Nodular gastritis	20 (3.0)	1 (1.0)	1 (0.5)	8 (4.0)	10 (5.7)
White marble appearance	80 (11.9)	0	9 (4.5)	31 (15.6)	40 (22.7)
Crack-like mucosa	67 (10.0)	0	4 (2.0)	62 (31.2)	1 (0.6)
Atrophic grading†					
C-0	333 (49.5)	59 (59.0)	118 (59.6)	74 (37.2)	82 (46.6)
C-1	133 (19.8)	5 (5.0)	14 (7.1)	90 (45.2)	24(13.6)
C-2	116 (17.2)	15 (15.0)	30 (15.2)	35 (17.6)	36 (20.5)
C-3	37 (5.5)	11 (11.0)	7 (3.5)	0	19 (10.8)
O-1	22 (3.3)	6 (6.0)	9 (4.5)	0	7 (4.0)
O-2	21 (3.1)	2 (2.0)	11 (5.6)	0	8 (4.5)
O-3	11 (1.6)	2 (2.0)	9 (4.5)	0	0
Pets					
No history	208 (30.9)	33 (33.0)	89 (44.9)	47 (23.6)	39 (22.2)
Dog	354 (52.6)	48 (48.0)	85 (42.9)	110 (55.3)	111 (63.1)
Cat	148 (22.0)	29 (29.0)	26 (13.1)	46 (23.1)	47 (26.7)
Pork offal ingestion					
No history	275 (40.9)	11 (11.0)	121 (61.1)	29 (14.6)	114 (64.8)
Sometimes	347 (51.6)	78 (78.0)	75 (37.9)	154 (77.4)	40 (22.7)
Often	51 (7.6)	11 (11.0)	2 (1.0)	16 (8.0)	22 (12.5)
Infection status					
*H. pylori* infection	24 (3.6)	7 (7.0)	3 (1.5)	6 (3.0)	8 (4.5)
Posteradication‡	256 (38.0)	35 (35.0)	72 (36.4)	61 (30.7)	88 (50.0)
No *H. pylori* infection	393 (58.4)	58 (58.0)	123 (62.1)	132 (66.3)	80 (45.5)

*Values are no. (%) except as indicated. Hospital A is in Hokkaido, B is in Tokyo, C is in Nagano, and D is in Okayama. †C-0, no atrophy; C-1 and C-2, mild atrophy; C-3 and O-1, moderate atrophy; and O-2 and O-3, severe atrophy. ‡Patients who had previous *H. pylori* eradication therapy.

Intragastric fluid from all patients was tested for NHPH infection. NHPH infections were found in 3% (20/673) of patients; *H. suis* infection was found in 70% (14/20) and non–*H. suis* NHPH infections in 30% (6/20) of those patients. Among the 20 patients, 15 were in the no *H. pylori* infection group, 3 in the *H. pylori* infection group, and 2 in the posteradication group. Diagnoses of the 3 NHPH-positive patients in the *H. pylori* infection group were made by using either the urea breath test, serum *H. pylori* antibody test, or endoscopic findings. However, we found those patients to be *H. pylori* negative by PCR targeting the *H. pylori*–specific region of the 16S rRNA gene (*21*). Therefore, those 3 patients were considered to be false positives for *H. pylori* because of their NHPH infection. The 2 NHPH-positive patients in the posteradication group had no history of *H. pylori* eradication; 1 of those patients was *H. pylori* negative by serologic and histologic analyses, and both showed *H. pylori*–negative status by PCR. Therefore, both patients were placed in the posteradication group according to endoscopic observations of NHPH infection and Kyoto classification of gastritis. After detecting NHPH species in intragastric fluid by PCR, patients were recategorized as follows: no *Helicobacter* infection, n = 378; posteradication, n = 254; *H. pylori* infection, n = 21; *H. suis* infection, n = 14; and non–*H. suis* NHPH infection, n = 6. The proportion of patients with *H. suis* infections was significantly higher in hospital C than in the other hospitals (p<10^−5^) (Table 2).

**Table 2:** Infection status of patients in 4 hospitals in study of NHPH infections, Japan, 2022*

Infection status	No. (%) patients					
	Total, n = 673		Hospital†			
			A, n = 100	B, n = 198	C, n = 199	D, n = 176
No *Helicobacter* infection	378 (56.2)		57 (57.0)	121 (61.1)	121 (60.8)	79 (44.9)
Posteradication‡	254 (37.7)		35 (35.0)	72 (36.4)	59 (29.6)	88 (50.0)
*H. pylori* infection	21 (3.1)		6 (6.0)	2 (1.0)	5 (2.5)	8 (4.5)
*H. suis* infection	14 (2.1)		0	1 (0.5)	13 (6.5)	0
Non–*H. suis* NHPH infection	6 (0.9)		2 (2.0)	2 (1.0)	1 (0.5)	1 (0.6)

*NHPH, non–*Helicobacter pylori* *Helicobacter*. †Hospital A is in Hokkaido, B is in Tokyo, C is in Nagano, and D is in Okayama. ‡Patients who had previous *H. pylori* eradication therapy.

### Comparison of Clinical Characteristics among Groups

**Figure 2:**
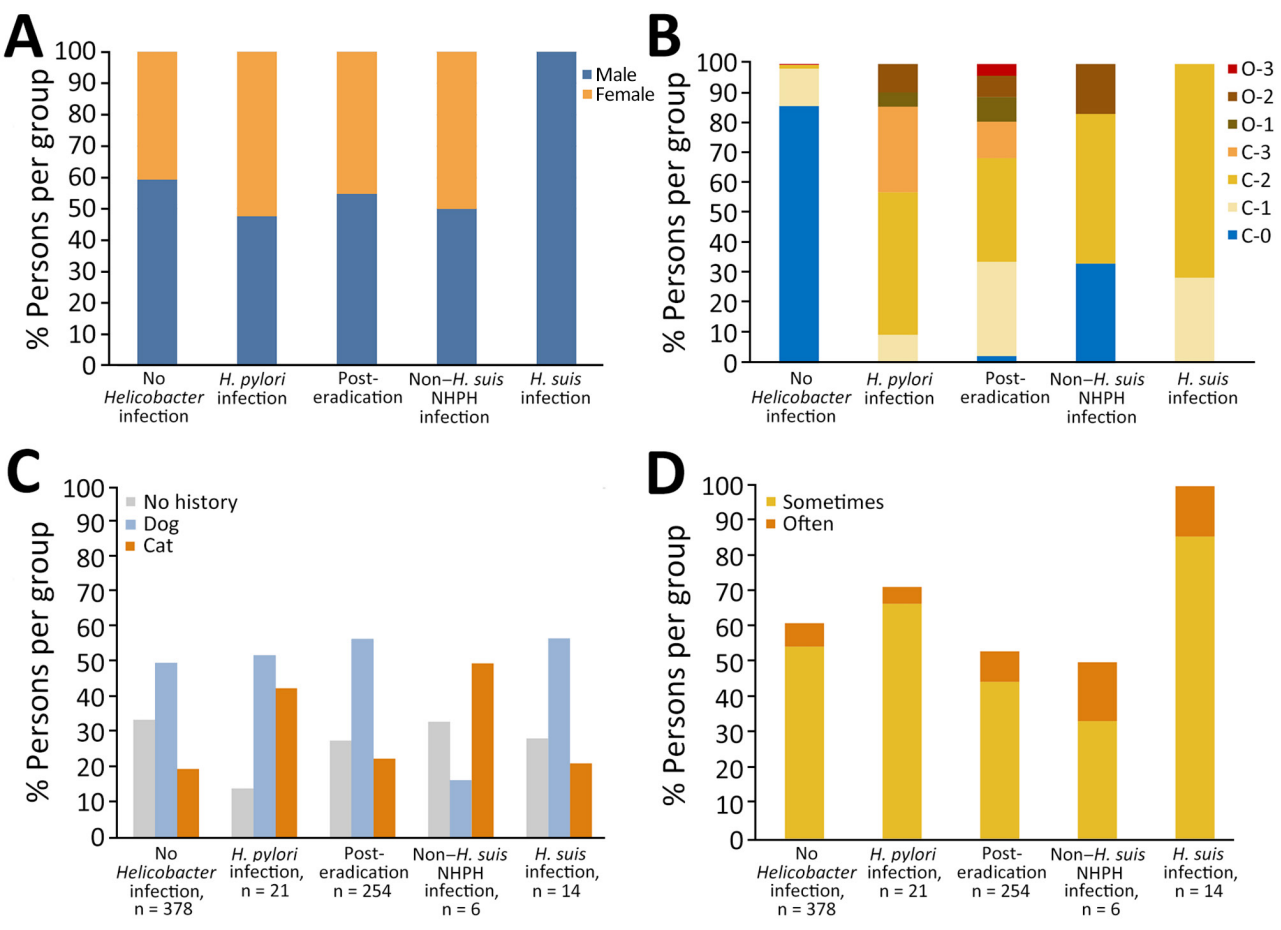
Comparison of patient groups stratified by gastric *Helicobacter* infection status in study of NHPH infections, Japan, 2022. A) Percentage of men and women in each group. All patients in the *H. suis* infection group were men. Prevalence of the male sex was significantly higher in the *H. suis* infection group than in the no *Helicobacter* infection (59.3%), *H. pylori* infection (47.6%), posteradication (54.7%), and non–*H. suis* NHPH infection (50.0%) groups. B) Percentages of patients categorized by each atrophic grade were compared between groups stratified by gastric *Helicobacter* infection status. In the *H. suis* infected group, 71.4% were C-2, with no higher grade of atrophy. C-0, no atrophy; C-1 and C-2, mild atrophy; C-3 and O-1, moderate atrophy; O-2 and O-3, severe atrophy. C) Percentages of patients who had no history of pets or who had a history of having pet dogs or cats. No significant differences were observed between the groups, although the proportion of patients with pet cats appeared higher (50%) in the non–*H. suis* NHPH infection group than in the no *Helicobacter* infection (19.8%) and *H. suis* infection (21.4%) groups. D) Patients in each infection group were subdivided into those who sometimes or those who often ingested pork offal. All patients in the *H. suis* infection group had a history of pork offal ingestion. Prevalence of patients with any history of pork offal ingestion was significantly higher in the *H. suis* infection group (100%) than in the no *Helicobacter* infection (61.1%; p = 0.0014), posteradication (53.1%; p = 0.0003), and non–*H. suis* NHPH infection (50.0%; p = 0.0175) groups. NHPH, non–*H. pylori Helicobacter*.

We compared clinical characteristics of patients in each of the recategorized groups (Table 3). The *H. suis* infection group consisted entirely of men (100%), which differed from the combined 4 groups not infected with *H. suis* (57% men; p = 0.0005) (Figure 2, panel A). When compared with the non–*H. suis* NHPH infection group (66.7%), the percentage of atrophic gastritis was 100% in the *H. suis* (p = 0.04) and *H. pylori* (p = 0.04) infection groups and 97.6% in the posteradication group (p = 0.01) (Table 3). The percentage of nodular gastritis was 42.9% for *H. suis*, 50.0% for non–*H. suis* NHPH, and 19.0% for *H. pylori* infection groups (p = 0.2 overall). The overall percentage of nodular gastritis among those 3 groups was 32%, which was significantly higher than that in the no *Helicobacter* infection (0%; p<0.0001) and posteradication (2.8%; p<0.0001) groups. In addition, a white marbled appearance (*18*) was observed in 78.6% and crack-like mucosa (*11*) in 85.7% of the *H. suis* infection group, indicating endoscopic characteristics of *H. suis* infection. The percentage of patients with at least 1 of the 2 endoscopic characteristics of NHPH infection was 92.9% (13/14) among the *H. suis*–infected patients, which was significantly higher than that among *H. pylori*–infected (28.6% [6/21]; p = 0.0003) and posteradication (27.6% [70/254]; p<0.0001) groups.

**Table 3:** Comparison of clinical characteristics between groups stratified by gastric *Helicobacter* species infection status in study of NHPH infections, Japan, 2022*

Characteristics	Gastric *Helicobacter* species infection status				
	No *Helicobacter* infection	*H. pylori* infection	Posteradication	Non–*H. suis* NHPH infection	*H. suis* infection
Total no. patients	378	21	254	6	14
Median age, y (range)	53 (29–84)	61 (35–88)	57 (30–84)	65 (44–91)	50 (35–66)
Sex					
M	224 (59.3)	10 (47.6)	139 (54.5)	3 (50.0)	14 (100.0)
F	154 (40.7)	11 (52.4)	115 (45.3)	3 (50.0)	0
Endoscopic findings					
Normal	317 (83.9)	0	5 (2.0)	0	0
Atrophic gastritis	53 (14.0)	21 (100.0)	248 (97.6)	4 (66.7)	14 (100)
Peptic ulcer	0	2 (9.5)	2 (0.8)	1 (16.7)	0
Gastric cancer	2 (0.5)	2 (9.5)	3 (1.2)	0	0
Nodular gastritis	0	4 (19.0)	7 (2.8)	3 (50.0)	6 (42.9)
White marble appearance	15 (4.0)	4 (19.0)	49 (19.3)	1 (16.7)	11 (78.6)
Crack-like mucosa	29 (7.7)	3 (14.3)	22 (8.7)	1 (16.7)	12 (85.7)
White marble appearance or crack-like mucosa	40 (10.6)	6 (28.6)	70 (27.6)	2 (33.3)	13 (92.9)
Atrophic grading†					
C-0	325 (86.0)	0	6 (2.4)	2 (33.3)	0
C-1	47 (12.4)	2 (9.5)	80 (31.5)	0	4 (28.6)
C-2	5 (1.3)	10 (47.6)	88 (34.6)	3 (50.0)	10 (71.4)
C-3	0	6 (28.6)	31 (12.2)	0	0
O-1	0	1 (4.8)	21 (8.3)	0	0
O-2	0	2 (9.5)	18 (7.1)	1 (16.7)	0
O-3	1 (0.3)	0	10 (3.9)	0	0
Pets					
No history	128 (33.9)	3 (14.3)	71 (28.0)	2 (33.3)	4 (28.6)
Dog	189 (50.0)	11 (52.4)	145 (57.1)	1 (16.7)	8 (57.1)
Cat	75 (19.8)	9 (42.9)	58 (22.8)	3 (50.0)	3 (21.4)
Pork offal ingestion					
No history	147 (38.9)	6 (28.6)	119 (46.9)	3 (50.0)	0
Sometimes	206 (54.5)	14 (66.7)	113 (44.5)	2 (33.3)	12 (85.7)
Often	25 (6.6)	1 (4.8)	22 (8.7)	1 (16.7)	2 (14.3)

*Values are no. (%) except as indicated. NHPH, non–*Helicobacter pylori* *Helicobacter*. †C-0, no atrophy; C-1 and C-2, mild atrophy; C-3 and O-1, moderate atrophy; and O-2 and O-3, severe atrophy.

The atrophic grade of patients with *H. suis* infection was mostly C-2, and no patient had an atrophic grade greater than C-2. Among *H. pylori*–infected patients, most had atrophic grades of C-2; however, some had O-1 (4.8%) and O-2 (9.5%) grades (Table 3; Figure 2, panel B).

Among the non–*H. suis* NHPH-infected patients, 50% (3/6) had a history of owning cats, which was higher than the 22% (145/667) observed among the other patients in the study. However, the difference was not statistically significant (p = 0.12) because of the small sample size of non–*H. suis* NHPH-infected patients (Figure 2, panel C). All (14/14) *H. suis*–infected patients had a pork offal ingestion history, a proportion that was significantly higher than that in the combined 4 groups not infected with *H. suis* (384/659; p = 0.0006) (Figure 2, panel D).

### Endoscopic Characteristics of Patients with NHPH Infection

We determined the type and site of gastritis and the presence or absence of RAC in the 20 NHPH-infected patients (Appendix Table). In each patient, gastritis was observed from the glandular border around the angulus to the pyloric antrum (Figure 1; Appendix Figure 1). Endoscopic gastritis results differed by NHPH species. The percentages of MARC gastritis were significantly different between the *H. suis*–infected (92.9% [13/14]) and non–*H. suis* NHPH–infected (33.3% [2/6]) groups (p = 0.014), whereas spotty redness was observed at similar rates in the *H. suis*–infected (35.7% [5/14]) and non–*H. suis* NHPH–infected (50.0% [3/6]) groups (p = 0.6). Gastritis of the pyloric canal was found in 50.0% (7/14) of the *H. suis* infection group, whereas it was absent in 83.3% (5/6) of the non–*H. suis* NHPH infection group, although the difference was not statistically significant (p = 0.3). RAC was absent in 1 patient who had petechial erythema spread over the entire gastric body.

### *H. suis* ELISA

**Figure 3:**
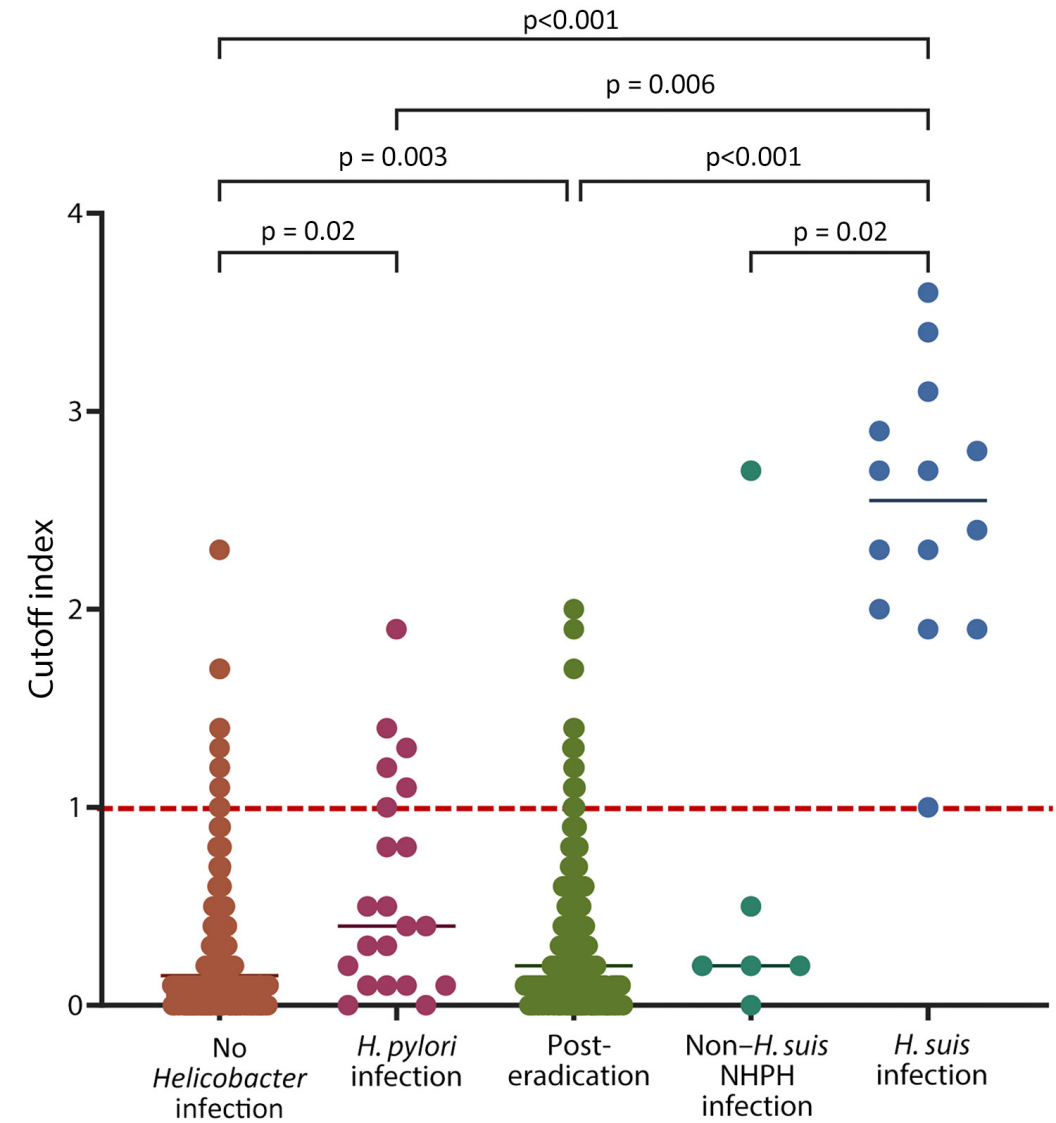
*Helicobacter suis* antibody titers according to gastric *Helicobacter* infection status in study of NHPH infections, Japan, 2022. Antibodies were measured by using ELISA. Gastric *Helicobacter* infection status was categorized into 5 groups. Horizontal lines within each group indicate the median value for that group. Red dotted line indicates the threshold for diagnosing *H. suis* infection, as previously described (*16*). p value comparisons are indicated above the groups. NHPH, non–*H. pylori Helicobacter*.

The COI for the *H. suis* ELISA was significantly higher for the *H. suis* infection group than for the other groups (Figure 3). All patients in the *H. suis* infection group were considered *H. suis* positive according to *H. suis* ELISA criteria (*16*). In the non–*H. suis* NHPH infection group, 1 (16.7%) patient was *H. suis* positive and 5 (83.3%) were *H. suis* negative, according to ELISA. We determined that 28.6% (6/21) of patients in the *H. pylori* infection, 7.1% (18/254) in the posteradication, and 3.4% (13/378) in the no *Helicobacter* infection groups were *H. suis* positive, according to *H. suis* ELISA. Among the 13 *H. suis*–positive patients in the no *Helicobacter* infection group, atrophic gastritis was found in 3 patients, suggesting the possibility of previous *H. suis* infection or false-negative results by PCR of intragastric fluid.

## Discussion

In this study of consecutive patients who underwent gastric endoscopy during medical checkups in Japan, the NHPH infection rate was 3%. NHPH infection rates of 0.25%–6.1% have been reported previously (*2*–*5*,*9*–*11*), indicating that rates among healthy persons are not much lower than among patients with gastric diseases. A 10-year study on NHPH infection rates in a single institution in Japan showed the infection rate increased from 1.3% to 3.35% (30 of 896 patients) in the last 2 years and 4 months of the study period because of increased recognition of characteristic endoscopic findings (*11*). Therefore, NHPH infections might be missed in many patients with gastric diseases. In our survey, the *H. pylori* infection rate was 3.6%, and 38% of patients had a history of *H. pylori* infection. The estimated *H. pylori* infection rate in persons born in Japan in 1968 was reported to be 37.5% (*22*). Considering the median age (55 years) of the patients in our study, our sample represents a major section of the population in Japan.

Gastric NHPH species have been found in patients with gastric diseases by using culture tests, including *H. suis* (*8*), which also infects pigs and monkeys, and *H. ailrurogastricus* (*23*), *H. felis* (*24*), and *H. bizzozeronii* (*25*,*26*), which infect cats and dogs. Other species that infect dogs and cats, such as *H. heilmannii* and *H. salomonis*, have also been detected by PCR in patients with gastric diseases (*2*–*5*,*9*–*12*). *H. suis* is the most prevalent NHPH species infecting humans in Japan and other countries, whereas other species infecting dogs and cats have also been detected in most studies (*2*–*5*,*9*–*12*). In this study, *H. suis* was the most prevalent NHPH species (14/20), consistent with previous reports in Japan (*4*,*11*).

All patients infected with *H. suis* in this study were men. The percentage of men in the group infected with non–*H. suis* NHPH species was 50%, similar (57.8%) to that in the entire tested study population. Other studies have also demonstrated a high prevalence of *H. suis* infections among men (*11*). Future studies should investigate the difference in prevalence of *H. suis* infection between men and women by focusing on aspects such as the transmission route of infection. The natural hosts of *H. suis* are pigs and monkeys, and pork meat is suspected to be a carrier of *H. suis* infection to humans. In this study, a relationship between the history of pork offal ingestion and *H. suis* infection was strongly suggested. Because a history of pork offal ingestion was found in only 40% of the no *Helicobacter* infection group, it was not always a risk factor for *H. suis* infection. However, hospital C, which had the highest percentage of *H. suis* infections, is located in an area where pork and pork offal are commonly eaten. A survey held in Japan in 2008 showed by using PCR that 74% of slaughtered sows were infected with *Helicobacter* spp. (*27*); however, updated surveys are needed to identify the current prevalence of *H. suis* infection in pigs.

Among NHPH-infected patients, 6 were infected with non–*H. suis* NHPH species. The non–*H. suis* NHPH species that infect dogs and cats are *H. felis*, *H. bizzozeronii*, *H. heilmannii*, *H. ailirogastricus*, *H. cynogastricus*, *H. baculiformis*, and *H. salomonis*. A survey of NHPH infections in dogs and cats in Japan indicated that 34.7% of dogs and 50% of cats were infected with NHPH species (*28*,*29*). Compared with other groups, 50% of patients infected with non–*H. suis* NHPH species had pet cats. Because not all patients had pets, pets might not be the only risk factor for non–*H. suis* NHPH infection. Multiple reports have suggested that non–*H. suis* NHPH species are transmitted from dogs and cats to humans (*23*–*26*), and studies demonstrating the genetic identity between strains from humans and pets will be needed to confirm that transmission. Species identification was not possible in this study because of the small number of bacterial cells in the samples. Further analysis using culture tests will be needed to identify non–*H. suis* NHPH species infecting humans.

No mixed infections of NHPH and *H. pylori* were observed; very low prevalences of mixed infections have been previously reported (*7*,*30*). Because NHPH infection was not observed in the posteradication group, *H. pylori* infection history might prevent NHPH infection, although further investigation is needed to clarify this possibility. Alternatively, NHPH might have also been eradicated by *H. pylori* eradication treatment. This study was conducted with patients who had undergone health checks in Japan; therefore, a substantial proportion of study participants had a history of *H. pylori* eradication therapy. Further analysis of a population with no history of *H. pylori i*nfection will be needed to clarify whether *H. pylori* infection prevents NHPH infection.

After we developed serologic tests for *H. suis* infection (*16*), we compared those results with PCR results. Serologic tests for *H. suis* infection revealed high detection sensitivity. Some patients in the no *Helicobacter* infection group tested positive in serologic tests for *H. suis* infection, and some of those patients had atrophic gastritis. Serologic tests will be useful to confirm PCR results and might help to detect overlooked *H. suis* infections. The serologic test for *H. suis* infection had low reactivity with the serum samples obtained from non–*H. suis* NHPH–infected patients. PCR remains the standard method for detecting NHPH infection, and a diagnostic method for non–*H. suis* NHPH infection is required. Some *H. pylori*–infected patients and patients with a history of *H. pylori* eradication showed positive serologic test results for *H. suis* infection, possibly because of cross-reactivity between *H. pylori* and NHPH species or NHPH infection might have been overlooked by PCR because of extremely low bacterial counts. Alternatively, the patients might have had a history of NHPH infection. Various possibilities exist; therefore, interviews regarding *H. pylori* eradication history are critical for accurately diagnosing NHPH infection by PCR and serologic tests. In addition to diagnostic testing, recognizing endoscopic characteristics of NHPH gastritis is essential. NHPH gastritis is antrum predominant and causes milder atrophy than *H. pylori* gastritis (*2*).

In this study, 94.1% (16/17) of patients with NHPH gastritis without *H. pylori* infection had RAC in the gastric corpus and had a Kimura-Takemoto classification of C-2 or lower, indicating that NHPH gastritis was localized within the angulus-antrum region and rarely extended to the gastric corpus (indicated by the presence of RAC). In addition, endoscopic gastritis of the pyloric canal was not observed in 60.0% of patients, suggesting that NHPH gastritis progresses from the angulus to the antrum, which differs from *H. pylori* gastritis, in which atrophy progresses from the antrum to the gastric body (Appendix Figure 2). In *H. pylori* gastritis, the incidence of gastric cancer increases as atrophy extends to the gastric body (*31*); however, in NHPH gastritis, atrophy does not extend to the gastric body, suggesting that gastric cancer might be less common. Because all asymptomatic NHPH-infected patients had MARC gastritis, nodular gastritis, or spotty redness similar to endoscopic gastritis, epidemiologic studies can determine whether NHPH gastritis is an etiopathogenic agent of gastric MALT lymphoma and peptic ulcers.

We found endoscopic characteristics of NHPH infection (i.e., MARC gastritis) in 93% of the *H. suis*–infected patients, which was more frequent than for *H. pylori*-infected patients (29%) and patients in the posteradication group (28%). Those findings were not observed in the non–*H. suis* NHPH group. Endoscopists should suspect NHPH infection, especially from *H. suis*, when NHPH-specific endoscopic characteristics are found in *H. pylori*–negative patients. Nodular gastritis was found in 42.9% (6/14) of the *H. suis*–infected group, similar to the rate in the non–*H. suis* NHPH infected group (50.0%), and was more frequent than in *H. pylori*–infected patients (19.0%), although the difference was not significant. Differences in the pathogenesis and endoscopic findings between *H. suis* and non–*H. suis* NHPH infections might become more apparent as the number of patients increases.

The first limitation of our study is that the study population was highly health conscious and had a large number of patients who had undergone *H. pylori* eradication therapy. Because a difference in the prevalence of *H. suis* infection was detected among the study areas, the population background must have had a substantial influence on the prevalence of NHPH species. Continued surveys targeting other areas in Japan and other countries can provide comprehensive information to elucidate the prevalence of NHPH infections. Second, because the participants underwent medical checkups, the age of the target group was relatively high; very few young persons were in the study population. Although endoscopy cannot be performed at all ages, a study using serologic testing for *H. suis* infection might be useful to determine *H. suis* infection status in young persons.

In conclusion, the prevalence of NHPH infection in patients who underwent gastric endoscopy during medical checkups in Japan was 3.0%, similar to that of *H. pylori* infection (3.1%). Whether all NHPH-infected patients need to receive an eradication regimen remains controversial, as does the contribution of NHPH infections to gastric MALT lymphoma and peptic ulcers. In regions with decreasing rates of *H. pylori* infections, it is crucial to differentiate NHPH infections. Our findings provide critical information on the prevalence and endoscopic characteristics of NHPH infections in Japan. NHPH infections are zoonotic. Our findings that pork offal ingestion is a risk factor for *H. suis* infections and having pet cats increases risk for non–*H. suis* NHPH infection can help prevent gastric *Helicobacter* diseases.

Appendix Additional information for prospective multicenter surveillance of non–*H. pylori Helicobacter* infections during medical checkups, Japan, 2022.
