# Supplementary material for: Prospective Multicenter Surveillance of Non–H. pylori Helicobacter Infections during Medical Checkups, Japan
Source: Emerg Infect Dis. 2025 Jun;31(6):1121–30. doi: 10.3201/eid3106.241315 (PMC12123907; doi:10.3201/eid3106.241315)
Supplement: Appendix — Additional information for prospective multicenter surveillance of non–H. pylori Helicobacter infections during medical checkups, Japan, 2022. [file 24-1315-Techapp-s1.pdf]

Article DOI: https://doi.org/10.3201/eid3106.241315
EID cannot ensure accessibility for supplementary materials supplied by authors. Readers who have difficulty accessing supplementary content should contact the authors for assistance.

Prospective Multicenter Surveillance of Non–*H. pylori Helicobacter* Infections during Medical Checkups, Japan, 2022

Appendix

Appendix Table. Endoscopic findings in NHPH-infected patients*

			Type of gastritis				Site of gastritis				RAC		
Patient	Age, y/sex	NHPH spp.	White marbled appearance	Crack-like mucosa	Nodular gastritis	Spotty redness	Pyloric canal†	Pyloric antrum	Glandular border around angulus	Gastric body, middle–upper	Pyloric canal, antrum, and angulus	Gastric body, lower–middle	Gastric body, upper
1	49/M	*Helicobacter suis*	+	+	+	+	–	+	+	–	–	+	+
2	65/M	*H. suis*	–	+	–	–	+	+	+	–	–	+	+
3	35/M	*H. suis*	+	+	+	–	+	+	+	–	–	+	+
4	51/M	*H. suis*	–	+	–	+	–	+	+	–	–	+	+
5	39/M	*H. suis*	+	–	–	+	–	+	+	+	–	–	–
6	39/M	*H. suis*	+	+	+	–	+	+	+	–	–	+	+

			Type of gastritis				Site of gastritis				RAC		
Patient	Age, y/sex	NHPH spp.	White marbled appearance	Crack-like mucosa	Nodular gastritis	Spotty redness	Pyloric canal†	Pyloric antrum	Glandular border around angulus	Gastric body, middle–upper	Pyloric canal, antrum, and angulus	Gastric body, lower–middle	Gastric body, upper
7	59/M	*H. suis*	+	+	–	–	–	+	+	–	–	+	+
8	47/M	*H. suis*	+	+	+	–	+	+	+	–	–	+	+
9	45/M	*H. suis*	+	+	+	–	+	+	+	–	–	+	+
10	37/M	*H. suis*	–	–	+	–	–	+	+	–	–	+	+
11	51/M	*H. suis*	+	+	–	–	+	+	+	–	–	+	+
12	59/M	*H. suis*	+	+	–	+	–	+	+	–	–	+	+
13	60/M	*H. suis*	+	+	–	–	–	+	+	–	–	+	+
14	66/M	*H. suis*	+	+	–	+	+	+	+	+	–	+	+
15	91/M	non-*H. suis*	+	–	–	–	+	+	+	+	–	–	–
16	75/M	non-*H. suis*	–	–	–	+	–	+	+	–	–	+	+
17	53/F	non-*H. suis*	–	–	+	–	–	+	+	–	–	+	+
18	66/F	non-*H. suis*	–	+‡	+	+	–	+	+	+	–	+	+
19	44/M	non-*H. suis*	–	–	–	+	–	+	+	–	–	+	+
20	63/F	non-*H. suis*	–	–	+	–	–	+	+	–	–	+	+

*NHPH, non-*H. pylori Helicobacter*; RAC, regular arrangement of the collecting venules; +, positive; –, negative.

†Site was within 3 cm of the pyloric ring,

‡Patient used proton pump inhibitor.

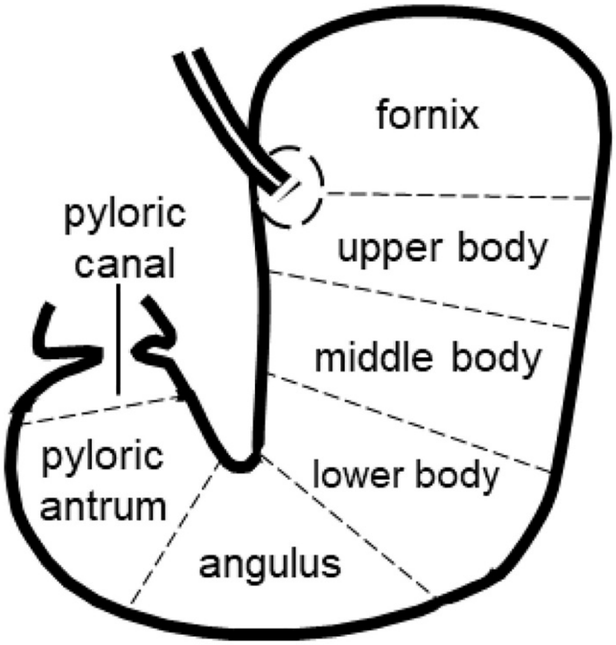

Appendix Figure 1. Definitions of stomach regions.

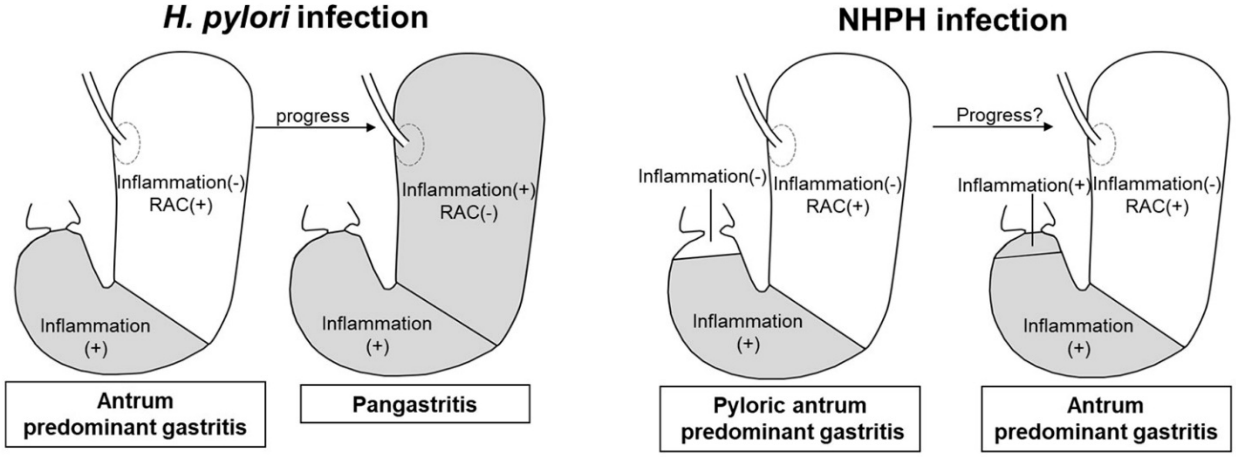

Appendix Figure 2. Differences in gastritis progression between *Helicobacter pylori* infection and NHPH species infections. NHPH, non-*H. pylori Helicobacter*; RAC, regular arrangement of the collecting venules; +, positive; –, negative.
